# Effect of Lactic Acid Bacteria on the Level of Antinutrients in Pulses: A Case Study of a Fermented Faba Bean–Oat Product

**DOI:** 10.3390/foods12213922

**Published:** 2023-10-26

**Authors:** Minna Kahala, Iida Ikonen, Lucia Blasco, Rina Bragge, Juha-Matti Pihlava, Markus Nurmi, Anne Pihlanto

**Affiliations:** Natural Resources Institute Finland (Luke), Production Technologies, Myllytie 1, FI-31600 Jokioinen, Finland; ikoneniida@outlook.com (I.I.); lucia.blasco@luke.fi (L.B.); rina.bragge@gmail.com (R.B.); juha-matti.pihlava@luke.fi (J.-M.P.); markus.nurmi@luke.fi (M.N.); anne.pihlanto@luke.fi (A.P.)

**Keywords:** lactic acid bacteria, faba bean, fermentation, antinutrients, galacto-oligosaccharides, vicine, convicine, organoleptic properties

## Abstract

The importance of cereals and pulses in the diet is widely recognized, and consumers are seeking for ways to balance their diet with plant-based options. However, the presence of antinutritional factors reduces their nutritional value by decreasing the bioavailability of proteins and minerals. This study’s aim was to select microbes and fermentation conditions to affect the nutritional value, taste, and safety of products. Single lactic acid bacteria (LAB) strains that reduce the levels of antinutrients in faba bean and pea were utilized in the selection of microbes for two starter mixtures. They were studied in fermentations of a faba bean–oat mixture at two temperatures for 24, 48, and 72 h. The levels of antinutrients, including galacto-oligosaccharides and pyrimidine glycosides (vicine and convicine), were determined. Furthermore, a sensory evaluation of the fermented product was conducted. Fermentations with selected single strains and microbial mixtures showed a significant reduction in the content of antinutrients, and vicine and convicine decreased by up to 99.7% and 96.1%, respectively. Similarly, the oligosaccharides were almost completely degraded. Selected LAB mixtures were also shown to affect the product’s sensory characteristics. Microbial consortia were shown to perform effectively in the fermentation of protein-rich materials, resulting in products with improved nutritional value and organoleptic properties.

## 1. Introduction

The global concern for environmental sustainability and food security, coupled with a focus on healthy eating and low costs, is driving the quest for new plant-based high-protein foods. Pulses such as beans, lentils, chickpeas, and peas are recognized worldwide as essential components of a healthy diet [1,2]. They also have several environmental benefits. For example, legume cultivation reduces greenhouse gas emissions, helps fix atmospheric nitrogen in the soil, and decreases the carbon footprint [3]. Pulses are excellent food sources for battling malnutrition and preventing chronic diseases because they are a good source of protein, dietary fiber, complex carbohydrates, vitamins, and minerals [4,5,6].

In recent years, there has been growing interest in combining various food sources to optimize the nutritional quality of meals. Among the combinations, cereals and pulses have emerged as a potent addition due to their complementary nutritional profiles. Pulses are well known for their high protein content, making them an excellent plant-based protein source. However, certain essential amino acids such as methionine and cysteine are frequently low or lacking in the amino acid composition of pulses [7,8,9]. For example, oats have a moderate protein amount but a more balanced amino acid composition. Oats have higher quantities of critical amino acids such as methionine and cysteine than pulses [10]. The combination of oats and pulses can, therefore, result in a better balance of essential amino acids, enabling a synergistic effect that enhances the overall quality of the protein.

Despite all their nutritional benefits, pulses also contain antinutritional factors such as phytic acid, protease inhibitors, lectins, and tannins that can reduce the bioavailability and absorption of important nutrients, harm digestion, or potentially have negative physiological effects [11,12]. Vicine and convicine are naturally occurring pyrimidine glycosides considered as antinutritional compounds that can cause favism, a disorder caused by a deficiency of the enzyme glucose-6-phosphate dehydrogenase (G6PD). Favism is characterized by the destruction of red blood cells, which causes anemia and other health problems. Boosting the intake of pulses and gaining the benefits of their use, therefore, depend on optimizing the nutritional value of pulses and avoiding the potential negative effects of antinutrients.

Pulses contain a wide range of factors that are relevant for gastrointestinal health. One of the compounds of interest is galacto-oligosaccharides (GOS), which are plant storage carbohydrates especially abundant in pulses. They are composed of α(1–6) linked galactose molecules bound to a disaccharide sucrose (one for raffinose, two for stachyose, and three for verbascose). Because humans do not produce galactosidase, these molecules are not hydrolyzed during human gastrointestinal digestion. They have, therefore, been linked to both antinutritional (flatulence) and prebiotic properties [13,14]. Fermentation offers a promising approach to improving nutrient absorption and reducing potential negative effects by decreasing the antinutritional compounds found in the pulses [15]. Through microbial action, fermentation can enzymatically degrade or modify antinutritional factors, thus, affecting the product’s nutritional value and contribution to human health. Additionally, the microorganisms responsible for pulse fermentation create enzymes that simplify complex lipids, proteins, and carbohydrates for easier digestion and absorption.

This study investigated the effect of fermentation with selected single lactic acid bacteria (LAB) strains on the levels of particular antinutrients in faba bean and pea, including GOS (galacto-oligosaccharides), vicine, and convicine, and the application of two selected microbial mixtures in the fermentation of a faba bean–oat mixture and their effect on the antinutrient content and sensory characteristics.

## 2. Materials and Methods

### 2.1. Plant Materials

The faba beans and peas used in this study were harvested in 2019 in Southwest Finland and purchased from Ypäjän Mylly, Finland. The oat flakes (Elovena^®^, Raisio Oyj, Raisio, Finland) were purchased from the local supermarket.

### 2.2. Microorganisms and Starter Culture Preparation

The lactic acid bacteria were isolates from plant-based materials from Luke’s and NMBU’s culture collections and commercial sources (Table 1). The fermentations were carried out with single LAB strains or with a mixture of three different strains (Table 2).

The lactic acid bacteria were propagated in de Man Rogosa and Sharpe (MRS, BD Difco, Franklin Lakes, NJ, USA) medium at 30 °C. For fermentations, LAB were cultivated overnight to a cell density of 8–9 log cfu/mL, after which the cells were harvested by centrifugation (7500 rpm, 3 min), washed, and resuspended in sterile tap water. The final concentration of the strains and microbial mixtures was adjusted to 6–7 log cfu/mL at the beginning of fermentation. Commercial strains were used, as recommended by the manufacturer (Sacco, Srl, Cadorago, CO, Italy).

### 2.3. Plant-Based Fermentations

#### 2.3.1. Pretreatment of Pulse Materials

Pretreatment protocols for single LAB strain fermentations in pea and faba bean. One hundred grams of faba bean or pea seeds were soaked in 500 mL water overnight at room temperature. The excess water was removed, and 200 g of soaked peas or faba beans were crushed (pulse 3 × 7 s) using a cook processor (KitchenAid Artisan Cook Processor, 5KCF0104, Kitchen Aid, MI, USA). Then, 300 g of water was added, and the mixture was heated (90–100 °C) in the cooking processor for 1h (faba bean) and 2.5 h (pea).

Pretreatment of faba bean–oat mixture. Faba bean was pretreated as described above. The oat mass was prepared by combining oat flakes (Elovena^®^, Raisio Oyj, Raisio, Finland) and water in a ratio of 14:86 (*w*/*w*) in a cook processor, heated to 95 °C, and boiled for 10 min. Pretreated faba bean mass and oat porridge were combined in a ratio of 70:30 *w*/*w*, and the final mass was divided into 120 mL batches for fermentation.

#### 2.3.2. Fermentations

Fermentation with single LAB strains. After pretreatment of the raw materials, the mixtures were cooled to room temperature, and an inoculum (isolates 1% inoculum and commercial strains as recommended by the manufacturer) was added to each batch. All the fermentations with single strains were performed at 30 °C for 48 h. Samples were collected from LAB fermentations and controls (heat processed) for analyses of vicine/convicine (faba bean) and GOS. After the fermentation, the pH was measured (SevenCompactTM S210, Mettler Toledo, Greifensee, Switzerland). All fermentations were performed in triplicate.

Fermentations in faba bean–oat mixture using two different microbial consortia, MIX31 and MIX33, as starters. Fermentations with the microbial mixtures were conducted in 100 mL batches in triplicates, and each LAB strain was added to the batches with a 1% inoculation. The fermentations were conducted at 30 °C and 37 °C. The control batch was pretreated and incubated similarly but without any starter. Samples were collected after 24 h, 48 h, and 72 h of incubation from each batch for pH and microbiological analyses and stored at −20 °C for further analysis, including galacto-oligosaccharides and vicine/convicine. All the fermentations were performed in triplicate.

### 2.4. Microbiological Analyses

The collected samples were homogenized (230 rpm, 30 s) using a Stomacher 400 Circulator (Seward, West Sussex, UK), and serial dilutions were prepared in Ringer solution (VWR, Radnor, PA, USA). The determination of *Enterobacteriaceae* was carried out by plating a diluted bacterial suspension on Violet Red Bile Glucose Agar (VRBGA, Oxoid, Hampshire, UK). The colonies were counted after incubation at 37 °C for 24 h. Lactic acid bacteria growth during fermentation was detected on MRSA (Oxoid, UK) after incubation in anaerobic conditions at 30 °C for 72 h.

### 2.5. Chemical Analyses

#### 2.5.1. Determination of Galacto-Oligosaccharides

For the determination of galacto-oligosaccharides, i.e., trisaccharide raffinose, tetrasaccharide stachyose, and pentasaccharide verbascose, in a fermented faba bean–oat mixture, the samples were prepared using a method modified from the protocol described by Ispiryan et al. [16]. Briefly, 1 mL of MeOH was added to a slightly thawed faba bean–oat sample (400 ± 20 mg), and the mixture was incubated for 5 min to deactivate enzyme activity. Next, 10 mL of ultrapure water was added to the sample tube, and the sample was homogenized by an Ultra Turrax homogenizer (T-25 digital, IKA, Staufen, Germany). The blade of the homogenizer was rinsed with 20 mL of purified water, and this was added to the sample. The sample was then placed in an ultrasonic bath for 10 min and then incubated in a water bath with agitation (80 rpm) at 80 °C for 1 h. After incubation, the sample was centrifuged (2500× *g*, 10 min), and the supernatant was transferred into a 100 mL volumetric flask. The remaining pellet was re-extracted (0.5 h) with 30 mL of ultrapure water in a hot water bath and centrifuged. Then, 200 µL of Carrez I and Carrez II solutions were added to the combined cooled supernatants to precipitate proteins. The volume was adjusted to 100 mL with ultrapure water, and the extract was mixed thoroughly. An aliquot of the sample was centrifuged at 2500× *g* for 10 min. An aliquot of the supernatant was further diluted with ultrapure water (1:1 *v/v*) and filtered through a 0.2 µm PTFE syringe filter (VWR International, Radnor, PA, USA) into an autosampler vial for analysis.

Oligosaccharides were analyzed with high performance anion-exchange chromatography with pulsed amperometric detection (HPAEC-PAD) (Dionex Integrion, Thermo Fisher Scientific, Waltham, MA, USA). The compounds were separated with a Dionex CarboPac PA210–4 µm column (2 × 150 mm) with a corresponding guard column using 12 mM KOH as the mobile phase at a flowrate of 0.2 mL/min and a temperature of 30 °C.

The standards of D-(+)-raffinose, stachyose, and verbascose (Sigma-Aldrich Burlington, MA, USA) were prepared in ultrapure water, and the calibration curves were found at a concentration range of 0.1–50 mg/L. The retention times (RT) of raffinose, stachyose, and verbascose were 10.1, 11.4, and 14.9 min respectively. The data handling was carried out with Chromeleon 7.2 software. The results were calculated as milligrams per gram of dry matter of the sample (DM). The dry matter content was determined by drying the sample at 102 °C for 15 h.

#### 2.5.2. Determination of Vicine and Convicine

The extraction method used in this study was modified from the method of Gutierrez et al. [17]. Briefly, the thawed sample mass (1 ± 0.06 g) was mixed with 10 mL of ultrapure water and stirred in a magnetic mixer for 1 h. The sample was then centrifuged (1740× *g*, 10 min), and concentrated HCl was added at a ratio of 1:100 (*v/v*) to an aliquot of the supernatant. After mixing, the sample was again centrifuged (1740× *g*, 10 min), and the supernatant was filtered through a 0.45 µm PTFE syringe filter (VWR International, Radnor, PA, USA) into an autosampler vial. Two technical replicates were prepared from each sample.

The samples were analyzed by Agilent 1100 series high-performance liquid chromatography with a diode array detector (HPLC-DAD) (Agilent, Santa Clara, CA, USA). To separate the compounds, a reverse-phase column Atlantis T3 (3.0 × 150 mm, 3 µm) (Waters, Milford, MA, USA) was used. The temperature of the column was 35 °C, and the sample injection volume was 5 µL. A gradient of 50 mM phosphate buffer (pH 2.4, solvent A) and methanol (solvent B) was used as a mobile phase at 0.3 mL/min. The gradient was as follows: 0–3 min solvent B 0%; 3–10 min 0–25% (solvent B); 10–19 min hold at 25% (solvent B); and 19–20 min back to 0% (solvent B). The post-run equilibrium time was 15 min.

The detection of vicine and convicine was performed at 280 nm, and the quantification by the external standard method, with vicine as a reference compound (Sigma-Aldrich). Aglycones of vicine and convicine, divicine, and/or isouramil were also tentatively identified by their UV spectra and quantified using the calibration curve of vicine and applying a molecular weight factor of 2.14. For identification purposes, a UV spectrum from 190 to 450 nm was recorded.

#### 2.5.3. Determination of Amino Acids

The analysis of the total amino acids, AA tot (peptide bound and free), was performed by Eurofins (Eurofins Scientific Finland Oy, Raisionkaari 55, 21201 Raisio, Finland) by the chromatographic method. For tryptophan, the determination was based on method EU 152/2009 (LC-FLD) [18]. For cysteine+cystine and methionine, oxidative hydrolysis, and for the remaining amino acids, acid hydrolysis was applied, and the analysis method was based on ISO 13903:2005 (IC-UV) [19].

### 2.6. Sensory Analyses

Tentative product development—overnight-oat-style product. An “overnight-oat—style” product with different flavors was developed as a product prototype. Two batches of faba bean–oat mixture were prepared as described above. One was inoculated with MIX31 and the other with MIX33, and they were fermented for 72 h at 30 °C. Both batches had two different flavors: berry (100% lingonberry purée (Bonne Juomat Oy, Lohja, Finland); dried berry mix (cane sugar, cranberry, blueberry, lingonberry, blackcurrant (Biokia^®^ Kiantama Oy, Suomussalmi, Finland)) and apple-cinnamon (apple jam (Saarioinen Oy, Tampere, Finland); and cinnamon (Meira Oy, Helsinki, Finland).

The product’s sensory properties were evaluated by conducting an affective test consisting of a ranking test, hedonic tests (scale 1 to 7), and a just-about-right (JAR) test. A specialist panel (*n* = 12, age 23–65, 10 females and two males) was introduced to six blind- coded samples; non-flavored and two flavored samples fermented with MIX31 or MIX33. The samples were presented in 4 cl plastic cups, and the evaluation was conducted in controlled sensory laboratory conditions. The panelists were asked to evaluate the pleasantness of the sample’s appearance, aroma, taste, and mouthfeel and its intensity of sourness and sweetness, as well as its overall pleasantness. Finally, the panelists were asked to compare the samples and rank them according to their preference.

### 2.7. Statistical Analyses

The fermentation process was carried out in three separate biological replicates, and each sample was analyzed twice. The results for all the analyses are shown as mean ± SD. For single strains, a Student’s t-test was applied for the independent unpaired samples.

For the mixtures, the data analysis was performed using a paired t-test in MS Excel 2008. In each sample, significant differences (*p* < 0.05) in the concentrations of the analyzed compounds between samples from selected fermentation time points were examined. To indicate significant distinctions, different superscript letters were assigned to the respective data points.

For the sensory analyses, the Shapiro–Wilk test was used to verify if the data followed a normal distribution. Principal component analysis (PCA) was applied as a sensory assessment tool to evaluate differences in the fermented food products, and *p*-values were determined using a one-way ANOVA and Tukey’s post hoc. The statistical analysis was carried out using different packages in R (vegan, rstatix), All *p*-values were adjusted for multiple hypothesis testing using the Benjamini–Hochberg procedure; adjusted *p* < 0.05 was considered significant.

## 3. Results and Discussion

### 3.1. Cultivations Using a Set of Single LAB Strains

**Figure 1:**
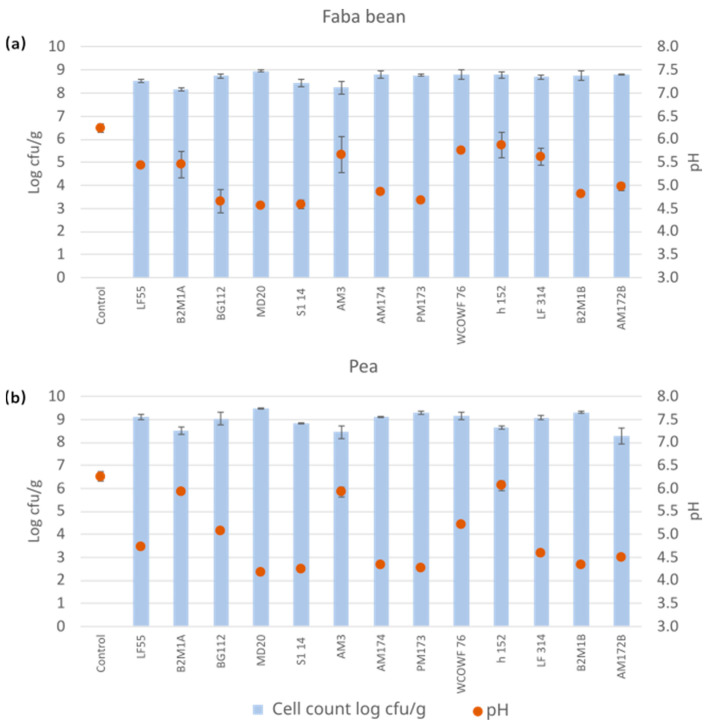
pH values and microbial counts on MRS agar after fermentation (48 h at 30 °C). A heated, unfermented sample served as a control for (**a**) faba bean and (**b**) pea. The blue bars indicate the cell count and orange dots the pH reached, both with the ±SD.

The success of the fermentation process was verified by the pH drop, determining the growth of LAB, and absence of enterobacteria. The counts of bacteria on MRS medium reached values of >8.0 log cfu/g after 48 h fermentation, indicating good growth of all the selected strains and showing the ability of the strains to grow well in the pulse-based media, both in pea and faba bean (Figure 1). Furthermore, pH drops were detected with pH varying from 4.57 ± 0.01 to 5.87 ± 0.27 in faba bean and from 4.18 ± 0.02 to 6.06 ± 0.10 in pea cultivations (Figure 1). The absence of enterobacteria in the fermented products indicated good hygienic quality, as the values of enterobacteria in all the samples were below the detection limit of <10 cfu/g.

### 3.2. Antinutrient Levels after Fermentation with Single LAB Strains

#### 3.2.1. Galacto-Oligosaccharides (GOS)

In the study, α-galacto-oligosaccharides (GOS) refers to the raffinose family oligosaccharides, which are the most common galacto-oligosaccharides in foods. Several microbes, including LAB, with the capacity to express α-galactosidase are known, and fermentation is, therefore, a potential alternative to reducing the concentration of GOS.

**Figure 2:**
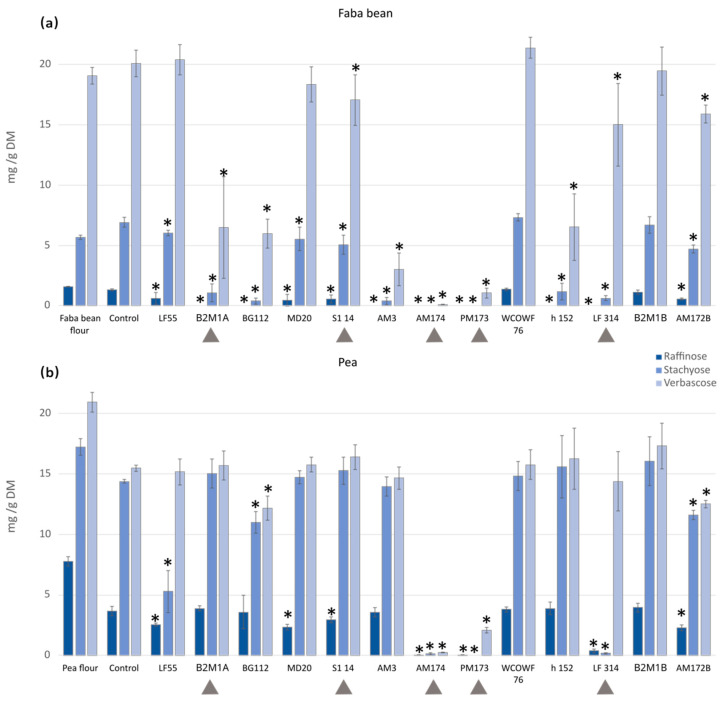
Level of GOS in (**a**) faba bean and (**b**) pea samples after being fermented with single strains for 48 h. A heat-treated unfermented sample served as a control. The arrows (▲) show the strains that were selected for starter mixtures. * Values significantly differ from the control (*p* < 0.05).

Prior to fermentation, verbascose was the most abundant GOS in faba bean, while stachyose and verbascose were the main GOS in pea (Figure 2). The cooking and autoclaving of the soaked beans were reported to improve the degradation, resulting in a decrease in GOS content of 50% or more [20]. In this study, pretreatment (soaking and cooking) reduced the content of raffinose in faba bean by 18%, and the total GOS was decreased in pea by 29% (the reduction was 52.7%, 16.4%, and 26.1% for raffinose, stachyose, and verbascose, respectively). The content of the GOS found in soaked, cooked, and non-fermented controls of faba bean was 1.3 ± 0.08 mg/g DM, 6.9 ± 0.4 mg/g DM, and 20.1 ± 2.3 mg/g DM for raffinose, stachyose, and verbascose, respectively. In pea, the content of raffinose, stachyose, and verbascose after soaking and cooking was 3.7± 0.4 mg/g DM, 14.4 ± 0.2 mg/g DM, and 15.5 ± 0.3 mg/g DM, respectively.

The fermentation process was shown to influence the amount and composition of GOS (Figure 2). The GOS reduction in fermented pulses was first studied with single strains (Figure 2a,b). The lowest levels of GOS were obtained in fermentations with the AM174 (*P. pentosaceus*) and PM173 (*Le. mesenteroides*) strains. AM174 reached levels under detectable limits in faba bean for raffinose and stachyose and very low levels (0.12 ± 0.01 µg/g DM) for verbascose, and similarly in pea, a significant reduction was observed, resulting in 0.03 ± 0.03, 0.13 ± 0.07, and 0.24 ± 0.03 µg/g DM for raffinose, stachyose, and verbascose, respectively. Similar observations were observed for PM173, as the levels obtained were below the detectable limits for raffinose and stachyose and 1.05 ± 0.43 µg/g DM for verbascose in faba bean, and 0.03 ± 0.03 µg/g DM for raffinose, undetectable for stachyose, and 2.09 ± 0.22 µg/g DM for verbascose in pea. The level of reduction was shown to depend on the raw material. AM174 and PM173 showed a significant reduction of all the three studied GOS in both pea and faba bean, while degradation using strains such as B2M1A, BG112, AM3, and h152 seemed more effective in faba bean.

Some reported data can be found on the effect of LAB in GOS content, although complete reductions of stachyose and verbascose are infrequent. Verni et al. [21] found that verbascose and stachyose could be completely degraded in the best performing fermentation conditions by *L. plantarum*. Furthermore, Rizzello et al. [22] reported complete degradation of raffinose in 48 h fermentation, as well as a significant decrease in stachyose and verbascose content from the *P. pentosaceus*, *Le. kimchi*, *Weissella cibaria,* and *W. confusa* LAB strains. Pea raffinose content was also shown to significantly decrease in LAB-fermented gelatinized pea flour dough [23].

#### 3.2.2. Vicine and Convicine

**Figure 3:**
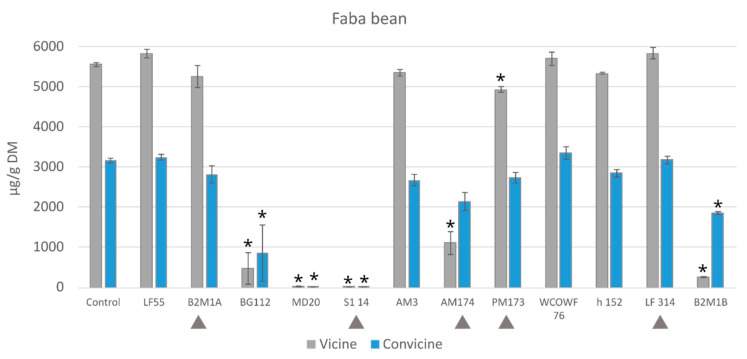
Reduction in vicine and convicine during 48 h fermentation by selected starter strains. A heated unfermented sample served as a control. The arrows (▲) show the strains that were selected for starter mixtures * Values significantly differ from the control (*p* < 0.05).

The finely ground mixture of raw materials, dry faba beans, and oat flakes was found to contain 4.5 ± 0.3 mg/g and 2.7 ± 0.1 mg/g DM of vicine and convicine, respectively. In comparison, the soaked and cooked non-fermented samples (controls) contained 4.5 ± 0.1 mg/g of vicine and 2.6 ± 0.2 mg/g of convicine on a dry weight basis. This indicates that the pretreatments employed in this study had a minimal impact on the concentration of vicine or convicine in faba beans. A set of selected single LAB strains was tested to decrease the level of these compounds during fermentation. The results of the single-strain fermentations verified that strains of S1 14 and MD20 were successful to completely degrade pyrimidine glycosides (>99%), whereas some of the other strains such as BG112 and B2M1B showed a high degradation of these compounds, with degradation values of 91.42% and 95.25% for vicine and 73.16% and 41.80% for convicine, respectively (Figure 3).

### 3.3. Cultivations with the Selected Microbial Mixtures

The microbial composition of the starter is known to affect the quality of the product. Fermentation is traditionally performed using microbial mixtures rather than single strains. In this study, the capability of single strains to degrade antinutrients was used to choose the strains for two different microbial mixtures. The aim was to include microbes acting in concert to produce the desired product characteristics. These two mixtures contained three different LAB strains (Table 2) and were applied in faba bean–oat processing. To select the strains for microbial mixtures for faba bean–oat fermentations, the characteristics of the single strains and their ability to grow and reduce the pH in a pulse-based medium, as well as reduce the level of antinutrients, GOS, and vicine/convicine, were considered. Based on the reduction of the GOS in both selected pulses, the highly efficient strains AM174 and PM173 and moderately efficient LF314, as well as B2M1A, which had good results in GOS reduction especially in faba bean, were selected. Furthermore, based on the reduction of vicine and convicine, another potential strain, S1 14, was added to one of the mixtures. All the strains selected for mixtures are well-known LAB strains, generally recognized as safe microorganisms, and frequently used in food fermentations. The mixtures contained both homo- and heterofermentative strains (Table 2). In the study, the effect of different fermentation conditions, including temperature and time, was tested to reduce the amount of antinutrients in the faba bean–oat mixture.

**Figure 4:**
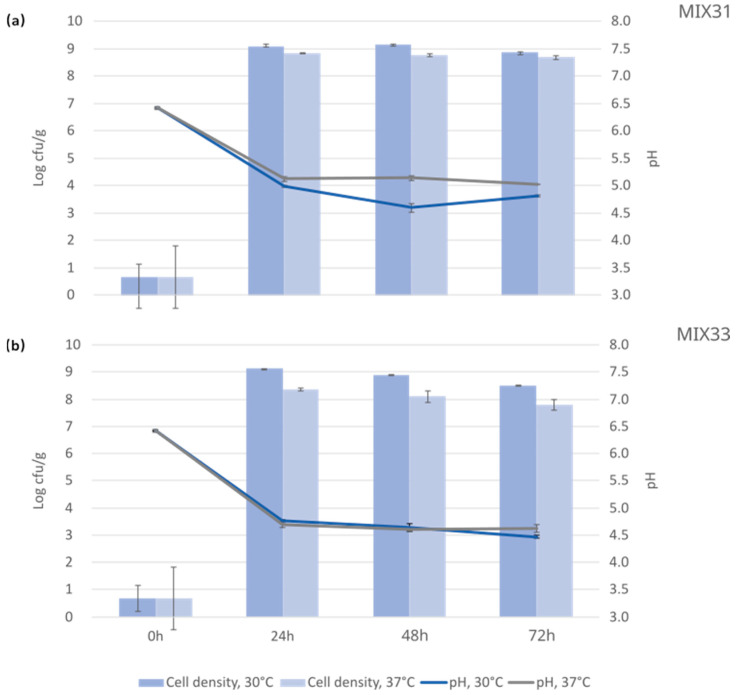
pH and cell density (log cfu/g) in the mixture of faba bean and oat before fermentation (0 h as a control) and after 24, 48, and 72 h fermentation for (**a**) LAB MIX31 and (**b**) MIX33 at 30 °C and 37 °C.

The selected LAB seemed to grow well in the faba bean–oat mixture at the studied temperatures, as a cell density of up to 8–9 log cfu/g was reached (Figure 4). Fermentation at 30 °C resulted in slightly higher cell densities than fermentation at 37 °C with both starter mixtures. Both starter mixtures effectively lowered pH, affecting the safety of the final product. Slight differences in the pH were obtained when the mixtures were compared, ranging at the end of fermentation between pH 4.8–5.0 for MIX31 and pH 4.4–4.6 for MIX33 (Figure 4).

### 3.4. Antinutrient Levels after Fermentation with Microbial Starter Mixtures

#### 3.4.1. Effect of the Microbial Mixtures on the Level of GOS

**Figure 5:**
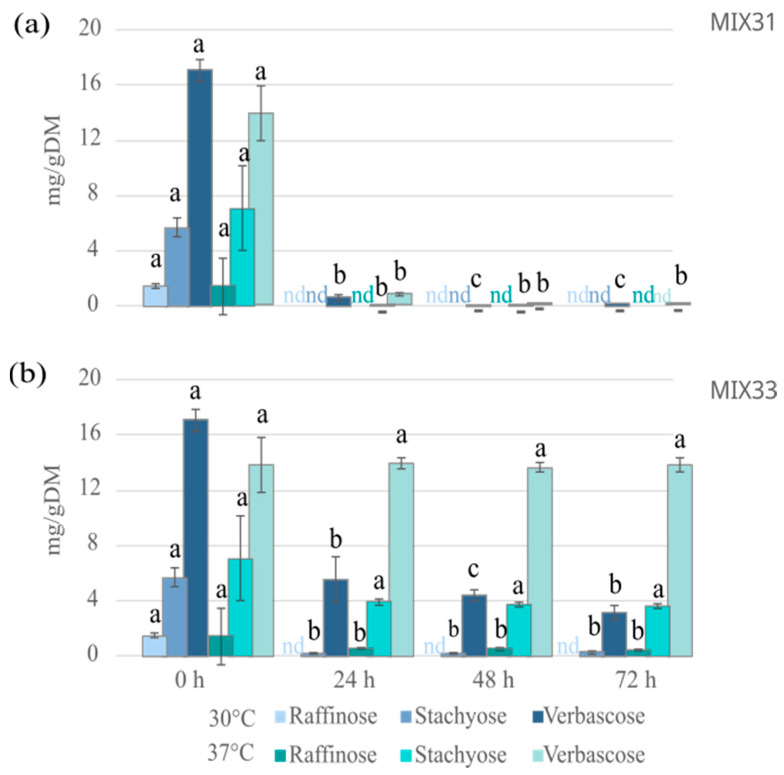
Galacto-oligosaccharide concentrations (mg/g DM) in faba bean–oat mixture fermented at 30 °C with (**a**) LAB MIX31 and (**b**) LAB MIX33 for 24 to 72 h. The bars represent the means of three independent experiments with a standard deviation (*n* = 3) as error bars. Statistically significant differences (paired *t*-test; *p*< 0.05) within the same temperature in each compound are shown by different letters (a–c). nd: not detected.

Regarding the GOS, both raffinose and stachyose disappeared completely, and a reduction of more than 96% in verbascose was already achieved after a 24 h fermentation with MIX31 at 30 °C (Figure 5). Raffinose was completely degraded in fermentation with MIX33, and the levels of stachyose and verbascose were already reduced by 96% and 70%, respectively, after 24 h of fermentation at 30 °C (Figure 5). A further reduction could be evidenced after 72 h fermentation, as stachyose was completely degraded, and verbascose was reduced by up to 99% using MIX 31, and stachyose by 96% and verbascose by 80% using MIX33. At 37 °C, an almost complete reduction of GOS was already obtained in fermentations with MIX31 at 24 h, reaching the maximum amount of degradation at 72 h (only 1% of verbascose left), while the reduction of GOS using MIX33 was unremarkable (Figure 5). These results are important when considering the development of pulse-based food products that cause less intestinal discomfort.

#### 3.4.2. Vicine and Convicine Levels after Fermentation with Microbial Mixtures

**Figure 6:**
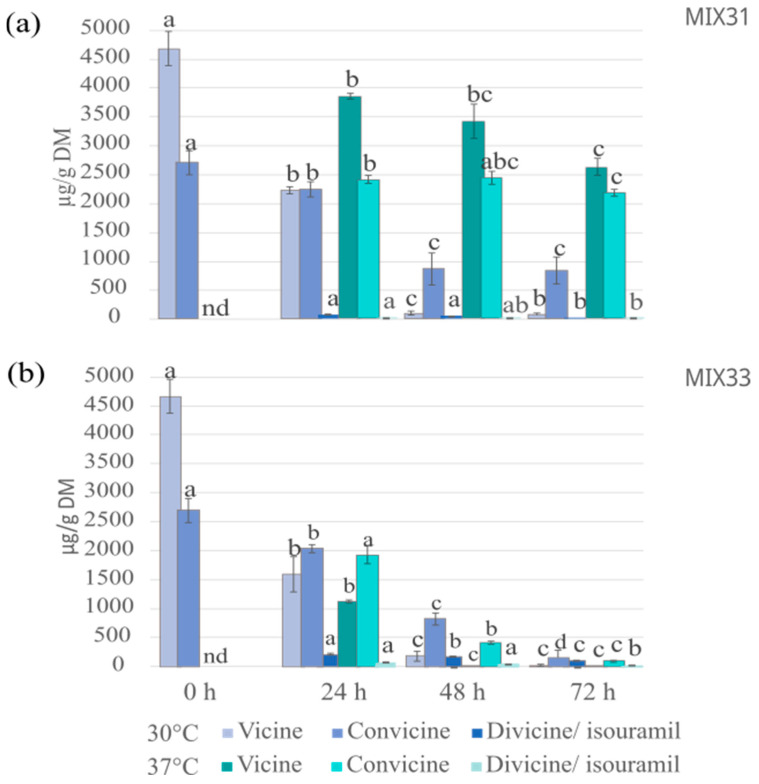
Concentration of vicine and convicine and their aglycones in a faba bean–oat mixture before and after fermentation with (**a**) MIX31 and (**b**) MIX33 at 30 °C and 37 °C. The bars are the means of three independent experiments ± standard deviation (*n* = 3) as error bars. Statistically significant differences (paired t-test; *p* < 0.05) within the same temperature in each compound are shown with different letters (a–d). nd: not detected.

The effect of microbial mixtures on the degradation of vicine and convicine in faba bean–oat mixture fermentations with MIX31 and MIX33 resulted in a reduction in levels of both vicine and convicine at both studied fermentation temperatures, with 30 °C proving slightly more effective, especially when using MIX31 (Figure 6). A 24 h fermentation with MIX31 at 30 °C resulted in a reduction of more than 50% in vicine, and after a 48 h fermentation, the residual vicine was only 2% (88 µg/g DM) (Figure 6). Similarly, the concentration of convicine decreased significantly (by 67%) after a 48 h fermentation. No major differences were observed in the concentrations compared with the levels after 48 h and 72 h fermentations. The levels of vicine and convicine aglycones, divicine, and isouramil were negligible after fermentation with MIX31. Compared to MIX31, MIX33 performed slightly better in reducing vicine and convicine. Considering the temperatures, higher reductions of the glycosides were obtained with MIX33 at 37 °C, resulting in reductions of more than 99% and 84% reduction in vicine and convicine, respectively, after a 48 h fermentation. Although vicine was not further reduced after 72 h, the convicine content was shown to be reduced by up to 96% during the extra day (Figure 6). Although MIX31 did not contain the strains with the best capacity to degrade vicine/convicine, the fermentation with this mixture at 30 °C indicates that a consortium with microbes acting together can achieve the desired result.

The elimination of vicine and convicine is targeted in faba bean breeding programs as their presence restricts the use of this pulse in foods [24]. Furthermore, ways to reduce vicine, convicine, and their aglycones from food have been studied by enzymatic treatments and fermentation, which led to a reduction of up to 85% and 47% in sourdough fermentation with the β-glucosidase active LAB strains, *L. plantarum* and *P. pentosaceus* [25]. As our study shows, Pulkkinen et al. [25] also observed that temperature had a slight effect on the level of hydrolysis of vicine and convicine. Fermentation with *L. plantarum* DPPMAB24W was successfully shown to reduce glycosides by more than 91% of the initial concentration of vicine and convicine [26] and almost complete degradation after 48 h of fermentation [27]. Furthermore, studies on reducing the glycoside concentrations in beans by other treatments, including heating such as frying, roasting, and boiling; continuous flow soaking in water; or soaking in acid, alkaline, or water have been reported [28,29].

### 3.5. Amino Acid Composition

Table 3 shows the amino acid (AA) composition and content of the samples. The content of individual AA, total AA (AA total), individual essential amino acids (EAA), and EAA total are expressed as g/kg DM. As expected, the results show that the addition of faba bean to oat improved and balanced the AA composition of oat with higher lysine content (oat 0.67 ± 0.01, faba bean 1.98 ± 0.05, and faba bean–oat mix control 1.57 ± 0.09 g/kg DM). We assumed that oat would increase the methionine and cysteine+cystine content in the final product, although only slight changes were evidenced. This may be because we used the oat flakes, and different pretreatment methods are known to decrease the EAA content in seeds [9].

### 3.6. Sensory Evaluation

**Figure 7:**
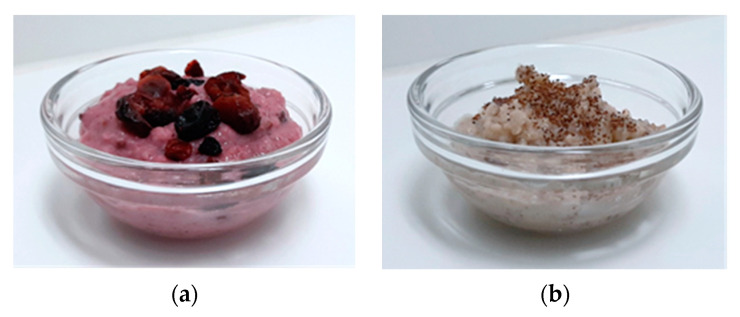
Fermented faba bean–oat mixture flavored with (**a**) berries and (**b**) apple and cinnamon.

The taste of the product is an important selection criterion for the consumer to select the product for consumption. LAB are known to produce compounds responsible for the flavor and texture of the fermented product. In our study, two microbial mixtures were used to produce a fermented fresh porridge-type product. The products were flavored with berries or fruit and spices (Figure 7). LAB typically produce a sour taste originating in the production of acids, which probably contain lactic and acetic acid, among others, in this study, as both homofermentative and heterofermentative strains are present in the microbial mixtures.

**Figure 8:**
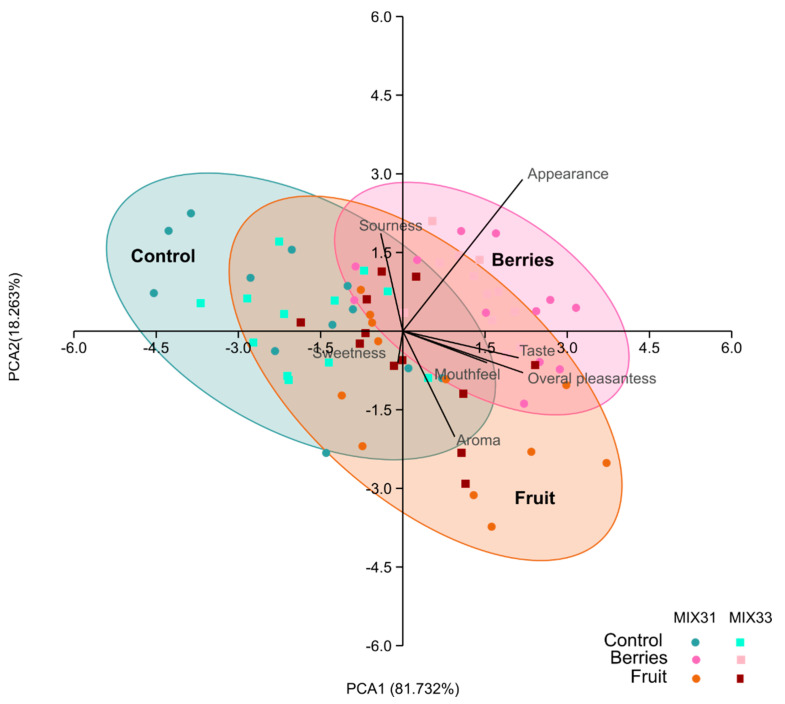
Principal component analysis biplot based on the sensory traits evaluated in the different samples prepared for the test (control, berries, and fruit, inoculated with MIX31 and MIX33). The variance explained by each principal component is given in the axis headings.

A sensory evaluation was performed using MIX31 and MIX33 to ferment samples, and two product concepts were developed (berries and fruit) to assess the impact of fermentation on the products’ flavor, as well as to estimate which flavors would either cover or support the sourness in the samples resulting from the lactic and acetic acid produced during fermentation. Flavored fermented samples received higher preference scores than non-flavored samples, especially for the appearance and mouthfeel of berry products, as well as aroma and mouthfeel of fruit products (Figure 8, Table 4). The overall pleasantness of the non-flavored samples was generally rated close to neutral (score 3.6, scale 1–7), i.e., neither pleasant nor unpleasant. Overall, flavored samples were preferred to unflavored samples, and according to overall pleasantness, the most highly ranked sample was the berry product fermented with MIX31 with a score of 5.3 (Figure 8, Table 4). When the unfermented samples were compared using the JAR test, the sample fermented with MIX31 was evaluated as more sour than MIX33. Products with fruity flavor were perceived less sour on average and evaluated as slightly towards “not sour enough.”

Based on the ANOVA, there were only a few statistically significant differences between the control and the different flavors (berries and fruit) (*p* < 0.05). Although there were some differences in taste and sourness between controls of MIX31 and MIX33, there were no significant differences for the total sensory characteristics for the MIX31 and MIX33 starter mixtures.

Along with improved nutritional quality, sensory properties are very important for increasing the consumption of faba beans. Fermented faba bean flour has previously been a focus, and it has been used as an ingredient in fortified sourdoughs [21,25,27] and pasta [30]. This study introduced a new approach to creating flavored fermented faba bean–oat snacks and assessing their sensory attributes. The results indicated that flavor enhancement improved the overall sensory experience. Despite variations in perceptions among the 12-persons panel, adding berries seemed to improve appearance, while the fruit-flavored product was perceived as less sour and more pleasantly sweet. These findings are preliminary due to the small sample size and structured tasting order. Future development should involve larger, trained panels for improved evaluation accuracy.

## 4. Conclusions

This work intended to promote fermented pulse-based products as a novel strategy for improving human nutrition and contributing to a healthy population by outlining the many advantages of fermented pulses. The study demonstrates the potential of specific microbial consortia to ferment pulses, reduce antinutrient levels, and enhance the nutritional value and safety of pulse-based foods. The results with these two microbial consortia clearly evidenced the importance of fermentation parameters, such as temperature and time, as well as the selection of microbes for the consortium, as the effects were seen in the final products characteristics, such as in the level of antinutrients and in the taste. By providing more options for balanced plant-based diets, especially for individuals following vegetarian or vegan diets heavily reliant on plant-based protein sources, these approaches can improve the bioavailability of essential nutrients. A decreased level of antinutrients can interfere with nutrient absorption or utilization and affect the bioavailability of certain minerals. The microbial consortia and the protocol developed were shown to have effective results in fermenting pulse-based raw materials, thus, offering new tools for product development within the food industry. Selecting high-quality pulses and developing proven microbial consortia are recommended for nutritious and sustainable dietary choices.

## Figures and Tables

**Table 1:** Single LAB strains selected for fermentations.

Strain	Species	Origin
LF55 (Sacco)	*Limosilactobacillus fermentum* (*L. fermentum*)	Sacco Srl, Italy
B2M1A (Luke)	*Levilactobacillus brevis (L. brevis)*	Isolated from pulses
BG112 (Sacco)	*Lactiplantibacillus plantarum* (*L. plantarum*)	Sacco Srl, Italy
MD20 (Luke)	*Lactiplantibacillus plantarum* (*L. plantarum*)	Isolated from cereals
S1 14 (Luke)	*Lactiplantibacillus plantarum (L. plantarum)*	Isolated from sourdough
AM3 (Luke)	*Weissella cibaria (W. cibaria)*	Isolated from pulses
AM174 (Luke)	*Pediococcus pentosaceus (P. pentosaceus)*	Isolated from pulses
PM173 (Luke)	*Leuconostoc mesenteroides (Le. mesenteroides)*	Isolated from pulses
WCOWF 76 (NMBU)	*Weissella confusa (W. confusa)*	Isolated from soybean/soybean–maize blends
h 152 (NMBU)	*Levilactobacillus brevis (L. brevis)*	Isolated from soybean/soybean–maize blends
LF314 (NMBU)	*Limosilactobacillus fermentum (L. fermentum)*	Isolated from soybean/soybean–maize blends
B2M1B (Luke)	*Lactococcus lactis (Lc. lactis)*	Isolated from pulses
AM172B (Luke)	*Lactococcus lactis* subsp. *lactis (Lc. lactis* subsp. *lactis)*	Isolated from pulses

**Table 2:** Strains and species of the lactic acid bacteria selected for starter mixtures MIX31 and MIX33.

Consortium	Strains	Genus and Species
MIX31	B2M1A	*Levilactobacillus brevis*
	AM174	*Pediococcus pentosaceus*
	LF 314	*Limosilactobacillus fermentum*
MIX33	S1 14	*Lactiplantibacillus plantarum*
	PM173	*Leuconostoc mesenteroides*
	B2M1A	*Levilactobacillus brevis*

**Table 3:** Amino acid composition of the individual cooked materials and fermented materials with MIX31 and MIX33, expressed in g/kg DM. Student’s t-test values between control and mixes showed no statistical differences on the amino acid profile after fermentation (*p* < 0.05).

	Faba Bean	Oat	Control FB:Oat	MIX31	MIX33
**Essential amino acids (EAA)**					
Cysteine + Cystine	0.3 ± 0.01	0.50 ± 0.01	0.4 ± 0.06	0.38 ± 0.02	0.41 ± 0.06
Histidine	0.84 ± 0.02	0.35 ± 0.01	0.67 ± 0.00	0.69 ± 0.00	0.72 ± 0.05
Isoleucine	1.26 ± 0.03	0.58 ± 0.01	1.01 ± 0.01	1.02 ± 0.03	1.05 ± 0.01
Leucine	2.31 ± 0.05	1.18 ± 0.02	1.90 ± 0.07	1.94 ± 0.11	1.97 ± 0.05
Lysine	1.98 ± 0.05	0.67 ± 0.01	1.57 ± 0.09	1.60 ± 0.13	1.64 ± 0.02
Methionine	0.21 ± 0.01	0.37 ± 0.01	0.24 ± 0.02	0.25 ± 0.01	0.23 ± 0.05
Phenylalanine	1.26 ± 0.03	0.81 ± 0.01	1.08 ± 0.00	1.13 ± 0.02	1.17 ± 0.05
Threonine	1.16 ± 0.03	0.57 ± 0.01	0.97 ± 0.05	0.96 ± 0.11	1.02 ± 0.02
Tryptophan	0.32 ± 0.01	0.23 ± 0.00	0.25 ± 0.02	0.26 ± 0.02	0.23 ± 0.01
Tyrosine	1.06 ± 0.03	0.62 ± 0.01	0.86 ± 0.86	0.88 ± 0.88	0.94 ± 0.94
Valine	1.41 ± 0.03	0.8 ± 0.01	1.19 ± 0.07	1.26 ± 0.03	1.26 ± 0.04
**Non-essential amino acids**					
Alanine	1.29 ± 0.03	0.75 ± 0.01	1.08 ± 0.06	1.13 ± 0.05	1.14 ± 0.05
Arginine	3.38 ± 0.08	1.15 ± 0.02	2.27 ± 0.36	2.07 ± 0.02	2.34 ± 0.06
Aspartic acid	3.34 ± 0.08	1.32 ± 0.02	2.68 ± 0.11	2.68 ± 0.23	2.82 ± 0.18
Glutamic acid	5.24 ± 0.12	3.26 ± 0.06	4.39 ± 0.27	4.52 ± 0.30	4.52 ± 0.13
Glycine	1.33 ± 0.03	0.79 ± 0.01	1.12 ± 0.07	1.18 ± 0.05	1.19 ± 0.04
Proline	1.26 ± 0.03	0.79 ± 0.01	1.08 ± 0.13	1.20 ± 0.01	1.26 ± 0.05
Serine	1.53 ± 0.04	0.83 ± 0.02	1.27 ± 0.06	1.23 ± 0.17	1.31 ± 0.05
Total AA	29.47	15.57	24.04	24.38	25.23
Total EAA	12.11	6.68	10.15	10.38	10.65

**Table 4:** Means and standard deviations of rates in the affective test (*n* = 12) in a balanced hedonic scale (scale 1–7; 1 extremely unpleasant, 5 neither pleasant nor unpleasant, 7 extremely pleasant) for appearance, aroma, taste, mouthfeel, and overall pleasantness, and a JAR scale (1–5) for sourness and sweetness. Significant differences between the rated samples based on a one-way ANOVA and Tukey’s post hoc test (*p* < 0.05) are indicated with letters (a–d).

	Affective Test					JAR Test	
	Appearance	Aroma	Taste	Mouthfeel	Overall Pleasantness	Sourness	Sweetness
Control MIX31	4.1 ± 0.2 ^a^	3.8 ± 0.3 ^a^	3.3 ± 0.4 ^a^	4.3 ± 0.4 ^abcd^	3.6 ± 0.3 ^a^	3.7 ± 0.3 ^ab^	
Control MIX33	4.2 ± 0.2 ^a^	3.8 ± 0.3 ^a^	3.6 ± 0.3 ^ac^	4.3 ± 0.3 ^a^	3.6 ± 0.3 ^a^	3.2 ± 0.3 ^abc^	
MIX31 berries	6.0 ± 0.2 ^b^	4.3 ± 0.3 ^abc^	5.3 ± 0.3 ^bc^	5.4 ± 0.2 ^bcd^	5.3 ± 0.3 ^b^	3.2 ± 0.2 ^ab^	2.8 ± 0.2
MIX33 berries	6.2 ± 0.2 ^b^	4.4 ± 0.3 ^abc^	4.8 ± 0.3 ^bc^	5.4 ± 0.2 ^bcd^	5.2 ± 0.2 ^b^	3.3 ± 0.2 ^ac^	2.6 ± 0.2
MIX31 fruit	4.4 ± 0.3 ^a^	5.5 ± 0.4 ^b^	4.9 ± 0.4 ^c^	5.4 ± 0.2 ^bcd^	5.1 ± 0.4 ^b^	2.6 ± 0.2 ^bc^	2.9 ± 0.2
MIX33 fruit	4.6 ± 0.3 ^a^	4.2 ± 0.3 ^ac^	4.6 ± 0.3 ^c^	5.1 ± 0.2 ^bcd^	4.9 ± 0.3 ^b^	2.7 ± 0.2 ^bc^	3.0 ± 0.3

## Data Availability

The data used to support the findings of this study can be made available by the corresponding author upon request.
